# Identification of introns harboring functional sequence elements through positional conservation

**DOI:** 10.1038/s41598-017-04476-0

**Published:** 2017-06-23

**Authors:** Michal Chorev, Alan Joseph Bekker, Jacob Goldberger, Liran Carmel

**Affiliations:** 1 Department of Genetics, The Alexander Silberman Institute of Life Sciences, Faculty of Science, The Hebrew University of Jerusalem, Edmond J. Safra Campus, Givat Ram, Jerusalem, 91904 Israel; 2 The Rachel and Selim Benin School of Computer Science and Engineering, The Hebrew University of Jerusalem, Edmond J. Safra Campus, Jerusalem, 91904 Israel; 3 Faculty of Engineering, Bar-Ilan University, Ramat Gan, 52900 Israel

## Abstract

Many human introns carry out a function, in the sense that they are critical to maintain normal cellular activity. Their identification is fundamental to understanding cellular processes and disease. However, being noncoding elements, such functional introns are poorly predicted based on traditional approaches of sequence and structure conservation. Here, we generated a dataset of human functional introns that carry out different types of functions. We showed that functional introns share common characteristics, such as higher positional conservation along the coding sequence and reduced loss rates, regardless of their specific function. A unique property of the data is that if an intron is unknown to be functional, it still does not mean that it is indeed non-functional. We developed a probabilistic framework that explicitly accounts for this unique property, and predicts which specific human introns are functional. We show that we successfully predict function even when the algorithm is trained on introns with a different type of function. This ability has many implications in studying regulatory networks, gene regulation, the effect of mutations outside exons on human disease, and on our general understanding of intron evolution and their functional exaptation in mammals.

## Introduction

Spliceosomal introns are one of the hallmarks of eukaryotic species, and inhabit genomes from the full spectrum of the eukaryotic domain. Many species, such as mammals, are intron-rich, harboring on average multiple introns per gene^1^. While many introns behave as neutral elements, many others has been shown to carry out a wide variety of cellular functions, and are involved in virtually every step of mRNA processing^2^. Here, we define intron function in the “causal role” sense, meaning that it contributes to cellular processes, and it need not necessarily adhere to the definition of “selected effect” functions, which are selected at the organism level^3^. For instance, introns participate in regulating transcription initiation, elongation, termination and timing^4–7^, in exporting mRNAs to the cytoplasm^8^, and in the quality control of mRNAs^9^.

This makes the identification of functional introns and the estimation of their abundance fundamental questions in functional genomics. However, it is not possible to detect functional introns by using traditional strategies such as looking for high sequence conservation. Intronic sequences – even of functional introns – rarely show elevated sequence conservation. One reason is that many intronic functions come about from short cis-regulatory elements that are hard to detect against a background of a very long non-functional intronic sequence, particularly when their location within the intron varies. A second reason is that not all intronic functions are sequence-dependent. Some intronic functions depend on the intron length rather than on its sequence. This is demonstrated by the E74 gene in *D. melanogaster*. The pre-mRNA of the predominant transcript of this gene is 60 kb long, but the mature mRNA is only 6 kb long. Thummel *et al*. measured the RNA polymerase II elongation rate on this gene and suggested that the time it takes to transcribe the introns generate delays that are crucial to the function of this gene during metamorphosis^10^. Other intronic functions depend on the position of the intron along the mRNA rather than on its sequence. This is the case, for example, in nonsense-mediated decay in mammals, where the splicing of introns sufficiently downstream of a termination codon marks the transcript for degradation^11, 12^. Still more intronic functions depend on the fact that splicing occurred along the transcript, regardless of which specific intron had been excised. This is the case, for example, in functions related to mRNA export and quality control^8, 13^.

As opposed to their generally poor sequence conservation, some introns show high conservation of their position with respect to the coding sequence, which is sometimes stable across billions of years^14, 15^. We conjectured that introns that harbor elements that are under purifying selection would experience a different selection regime than other introns, and therefore display distinct evolutionary characteristics that are reflected in lower loss rates and higher positional conservation. In a previous paper, we provided support to this conjecture. We identified 503 human introns that harbor miRNAs and snoRNAs. We used several features to quantify their level of positional conservation, compared them to 5,603 human introns that are not known to harbor functional elements, and demonstrated that indeed introns harboring functional elements show unique evolutionary patterns^16^. Specifically, they tend to be of ancient origin (otherwise their function would not have impacted their evolutionary pattern) and to show elevated levels of positional conservation.

Here, we developed a statistical framework to predict which introns harbor functional elements. First, we examined the characteristics of functional introns whose function originates from an entirely different class of functional elements. To this end, we used introns that harbor transcription factor binding sites (TFBS) for which we have functional evidence. We found that these introns show almost identical characteristics to introns that harbor small RNAs, thus demonstrating that high positional conservation is a universal property of such functional introns, regardless of the particular element embedded within their sequence. Based on these universal characteristics, we have devised a statistical model that computes the probability of each human intron to be functional in the sense that it harbors a functional element. Our statistical framework is unique in that it accounts for the inherent uncertainty in labeling an intron as non-functional. Whereas there is a high certainty that an intron tagged as functional is indeed so, many introns whose function is not known and are thus tagged as non-functional, may in fact be functional. Our approach explicitly models this uncertainty, and provides estimates to its magnitude.

## Results

### Gene architecture data sets of functional introns

In a previous work we identified 391 sets of orthologous transcripts across 28 species, in which the human transcript harbors at least one functional intron, and where the function stems from embedded miRNA or snoRNA elements^16^.

The 28 species comprise of: Six mammals (*Homo sapiens, Pan troglodytes, Pongo abelii, Mus musculus, Monodelphis domestica, Ornithorhynchus anatinus*); a bird (*Gallus gallus*); a lizard (*Anolis carolinensis*); an amphibian (*Xenopus tropicalis*); two fish (*Danio rerio*, *Gasterosteus aculeatus*); and one member of Cionidae (*Ciona savignyi*), which together make the representatives of Chordates in this data set. In addition, our data set includes three protostomes (*Drosophila melanogaster*, *Caenorhabditis elegans, Schistosoma mansoni*), which, together with the chordates, are all bilaterians. Furthermore, our data set contains four fungi (*Ustilago maydis, Saccharomyces cerevisiae, Neurospora crassa, Schizosaccharomyces pombe*). Other Eukaryotic subgroup include Viridiplantae (consisting of *Vitis vinifera, Arabidopsis thaliana, Oryza sativa, Physcomitrella patens*); two representative of Stramenopiles (*Phaeodactylum tricornutum, Phytophthora infestans*)*;* a single Alveolate (*Plasmodium falciparum*); a single Amoebozoan (*Dictyostelium discoideum*); and a single Euglenozoan (*Leishmania major*). This list of species may not be ideal, as it includes lineages that had gone through massive intron loss (like *S. cerevisiae* and *U. maydis*) and lineages that harbor a small number of introns (like *L. major* and *P. falciparum*). In addition, some lineages are connected to the rest through long branches in the phylogenetic tree, presenting some challenges to the interpretation of the results. On the other hand, these species were selected based on the fact that their genes have known lists of orthologs, allowing for large-scale comparative genomics. Indeed, the species included in this study were shown to provide meaningful information on evolutionary trends of intron evolution^16^.

Here, in order to test whether functional introns show characteristics that are independent of the specific function, we have identified many more human functional introns, where the function does not come from embedded small RNAs, but rather from the presence of transcription factor binding sites (TFBS). The mere presence of a TFBS within an intron is not an indication of functionality^3, 17^, as TFBS are greatly over-represented in the genome and only a small fraction of them has a true biological significance^18, 19^. To avoid this, we only considered TFBS that coincide with human active genomic segments as mapped by the ENCODE project^20–23^. We have created two data sets: Dataset 1 is a direct extension of our previous data set, consisting 771 sets of orthologous transcripts across 28 species. Of these, in 404 sets the human transcript contains at least one functional intron which harbors a miRNA, a snoRNA or a TFBS. In 367 sets, no intron along the human transcript is documented as functional. Dataset 2 consists of additional 8,661 sets of orthologs across the 28 species, in which the human transcript contains at least one functional intron harboring a TFBS.

**Figure 1:**
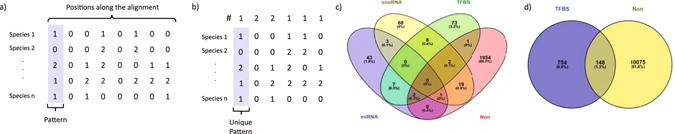
Data preparation. **(a)** Ternary representation of gene architectures within a set of orthologous transcripts. **(b)** The data from (a), represented by the unique patterns in the data and their number of occurrences. **(c)** Venn diagram showing the functions overlaps between functional and non-functional introns in Dataset 1. **(d)** Venn diagram showing the functions overlaps between functional and non-functional introns in Dataset 2. Venn diagrams were produced using Venny ^43^.

For each set of orthologs we generated multiple sequence alignment of the orthologous transcripts at the mRNA level. Upon this multiple sequence alignment we had mapped the gene architecture using a ternary representation, where 1 marks the last nucleotide of an exon, 0 marks all other nucleotides, and 2 marks a lack of ortholog or a gap. Each column in the ternary representation is called a pattern because it shows the intron presence-absence configuration at that position across the 28 species (Fig. 1a). We looked only at patterns that include at least a single 1, namely at patterns that correspond to an intron position in at least one species. Dataset 1 consists 9,011 patterns (an average of 11.7 patterns per set of orthologs), and Dataset 2 consists 44,122 patterns (an average of 5.1 patterns per set).

As long as introns are assumed to evolve independently of each other, two positions with the same pattern hold the same information on the underlying evolutionary processes. Therefore, pattern information in a data set can be compactly summarized by a list of all unique patterns, together with the number of times each unique pattern appears in the data set (Fig. 1b). We have labeled each unique pattern as belonging to one of three groups. If in all occurrences of a unique pattern the human intron is associated with a function (of whatever type), it was labeled as functional. If no function was associated with the human intron in any occurrence of a unique pattern, it was labeled as non-functional. Otherwise, when both functional and non-functional introns share the same unique pattern, it was labeled as partially functional (Fig. 1c,d). Finally, as function is only examined for human introns, the analysis was restricted to patterns that represent an intron in human. In total, Datasets 1 and 2 consist of 2,209 and 10,977 unique patterns, respectively (Table 1).

**Table 1:** Unique patterns composition in Datasets 1 and 2.

Function Type	Dataset 1	Dataset 2
miRNA-bearing	53 (2.4%)	0
partial miRNA-bearing	11 (0.5%)	0
snoRNA-bearing	99 (4.5%)	0
partial snoRNA-bearing	22 (1.0%)	0
TFBS-bearing	88 (3.98%)	1,123 (10.2%)
partial TFBS-bearing	5 (0.2%)	188 (1.7%)
Functional	222 (10.05%)	754 (6.87%)
Non-functional	1,954 (88.45%)	10,075 (91.78%)
Partially functional	33 (1.5%)	148 (1.35%)

The ‘Functional’ row is the union of all three functions, counting unique patterns that are functional. Some unique patterns are associated with more than a single function, leading to the fact that the number of functional unique patterns (222) is less than the sum of miRNA-, snoRNA- and TFBS-bearing unique patterns.

### Introns that harbor functional elements display common characteristics

In a previous work, we examined the effect of many pattern-characterizing features. Our initial list consisted of 48 features, but features that were highly associated with other features were removed. This process led to a final list of 13 features that represent diverse properties of the intron such as its antiquity, how its evolutionary pattern resembles that of other introns, the level of its positional conservation, and its position along the coding sequence (Table 2)^16^. Notably, these features do not include any gene-specific properties like expression level, as the same pattern can be present in multiple genes.

**Table 2:** Description of the pattern-characterizing features.

Feature	Description
LOG_LIKE	The log-likelihood of the pattern, computed by EREM^44, 45^
ONES_KNOWN	The ratio of 1’s in the pattern, excluding missing data
SANKOFF_G1L3	The total cost of the most parsimonious evolutionary process explaining the pattern, assuming that loss costs three times as much as gain
SANKOFF_G3L1	The total cost of the most parsimonious evolutionary process explaining the pattern, assuming that loss costs three times less than gain
IN_AMPHIBIAN	Binary value, equals 1 if the pattern contains at least one intron in amphibians
IN_FISH	Binary value, equals 1 if the pattern contains at least one intron in fish
IN_BIRD	Binary value, equals 1 if the pattern contains at least one intron in birds
IN_FUNGI	Binary value, equals 1 if the pattern contains at least one intron in fungi
IN_PLANT	Binary value, equals 1 if the pattern contains at least one intron in plants
IN_PROTIST	Binary value, equals 1 if the pattern contains at least one intron in protists
LCA_AGE	The age of the last common ancestor of all introns in the pattern, as inferred by Dollo parsimony
MED_POSITION	Median of the number of bases between the CDS start and the exon-exon junction
MED_REL_POSITION	Median of the number of bases between the CDS start and the exon-exon junction, divided by the total CDS length

Analysis of the 13 features for introns that harbor miRNAs and snoRNAs revealed some characteristic properties of these introns: First, their associated patterns have low likelihood, suggesting that their evolutionary history is different than that of the bulk of introns. Second, they show high positional conservation across metazoans. In particular, they have low intron loss rate even compared to the generally low loss rate in metazoans. Third, their origin is ancient, and usually predates the origin of metazoans. Indeed, as the identification of functional introns relies on their unique evolutionary histories, we do not expect to be able to identify introns which have recently gained their function^16^.

We started the analysis by examining these 13 features on Dataset 1. First, we compared the values of each feature across miRNA/snoRNA-bearing and -lacking patterns using Bonferroni-corrected t-test (Table 3) and Mann-Whitney U-test (Supplementary Table S1). Indeed, functional patterns display the same unique evolutionary characteristics that we observed before. These include lower average values of the LOGLIKE feature (−20.33 vs. −15.42, P = 1.6·10^−6^, t-test); greater antiquity, reflected by high values of LCA_AGE (1,134 vs. 956 MYA, P = 0.029) that measures the age of the last common ancestor of all species that harbor the intron; and a strong inclination to reside closer to the 5′ UTR (744 vs 1,531 nt, P = 1.8·10^−9^).

**Table 3:** Mean of the 13 features over TFBS-, miRNA- and snoRNA- bearing and lacking unique patterns. Median values can be found in the supplementary material and lead to the same conclusions.

	miRNA and snoRNA			TFBS in Dataset 1			TFBS in Dataset 2		
	Bearing	Lacking	P-value (t-test)	Bearing	Lacking	P-value (t-test)	Bearing	Lacking	P-value (t-test)
LOGLIKE	−20.33	−15.42	1.64 × 10^−6^	−26.94	−15.26	4.82 × 10^−22^	−23.53	−16.89	1.33 × 10^−63^
ONES_KNOWN	0.49	0.52	0.06	0.49	0.52	~1	0.55	0.55	~1
SANKOFF_G3L1	4.44	3.90	7.67 × 10^−6^	4.96	3.89	3.79 × 10^−14^	4.36	3.83	4.03 × 10^−34^
SANKOFF_G1L3	3.03	2.46	2.3 × 10^−4^	4.07	2.43	7.5 × 10^−21^	3.55	2.49	7.09 × 10^−71^
IN_AMPHIBIAN	0.89	0.89	~1	0.76	0.90	2.98 × 10^−4^	0.83	0.92	3.98 × 10^−14^
IN_FISH	0.93	0.91	~1	0.91	0.91	~1	0.90	0.91	~1
IN_BIRD	0.79	0.80	~1	0.62	0.81	1.99 × 10^−4^	0.64	0.8	2.17 × 10^−23^
IN_FUNGI	0.12	0.06	0.07	0.17	0.06	5.9 × 10^−4^	0.23	0.1	2.95 × 10^−28^
IN_PLANT	0.20	0.14	0.18	0.34	0.13	4.14 × 10^−7^	0.25	0.18	4.01 × 10^−5^
IN_PROTIST	0.12	0.09	~1	0.33	0.08	9.29 × 10^−14^	0.25	0.13	7.5 × 10^−20^
LCA_AGE	1134.30	955.91	2.92 × 10^−2^	1385.21	950.42	7.31 × 10^−8^	1280.45	1049.88	1.38 × 10^−14^
MED_REL_POSITION	0.49	0.51	~1	0.14	0.52	1.4 × 10^−38^	0.17	0.51	5.1 × 10^−257^
MED_POSITION	743.534	1531.25	1.75 × 10^−9^	265.77	1520.79	1.21 × 10^−14^	273.63	1084.13	3.15 × 10^−95^

To test whether introns with a completely different function have similar characteristics, we repeated the above comparison, contrasting TFBS-bearing patterns with patterns associated with a human intron not known to be functional (Table 3 and Supplementary Table S1). We found that introns harboring TFBS show very similar properties to introns that harbor small RNAs. Namely, they too have lower LOGLIKE values, prominent presence within different eukaryotic groups, and lower loss rates. More specifically, the average log-likelihood of TFBS-bearing patterns is −26.94, which is significantly lower than the average for TFBS-lacking patterns, which is −15.26 (P = 4.82·10^−22^, t-test). Moreover, introns that harbor TFBS in humans tend to be found also outside of metazoans: in fungi (0.17 vs. 0.061, P = 5.9·10^−4^), in protists (0.33 vs. 0.084, P = 9.3·10^−14^), and in plants (0.34 vs. 0.084, P = 9.3·10^−14^). This suggests the plausible evolutionary scenario by which these introns became functional much earlier than the birth of metazoans. It should be emphasized that it does not mean that the same TFBS populated these intron for such a long period, as TFBS turnover rate is known to be high^24^. Rather, different elements from within the same intron may have similar functions, keeping the intron functional. This is further supported by the values of the feature LCA_AGE. The average LCA_AGE of TFBS-bearing introns is significantly higher than that of TFBS–lacking patterns (1,385 vs. 950 MYA, P = 7.3·10^−8^), but also than that of miRNA- and snoRNA-bearing patterns (1,134 MYA, P = 0.015). Also in common to all functional introns is that both SANKOFF_G3L1 and SANKOFF_G1L3 are higher, which is again a result of their antiquity, providing them with more time to accumulate evolutionary events.

All types of functional patterns have a higher tendency to reside closer to the 5′-side of the gene (Table 3). This tendency is more pronounced for TFBS-bearing patters (median position 265 nucleotides from the CDS start), than for patterns that harbor small RNAs (median position 743 nucleotides from the CDS start), which is not surprising given that TFBS are oftentimes found at the beginning of the gene^25^. In a previous work we showed that this tendency for introns harboring small RNAs is fully explained by the fact that first introns tend to be longer^26^. In order to test whether this effect also explains the proximity of TBFS-bearing introns to the CDS start, we conducted a permutation test. To this end, we assumed as a null hypothesis that a functional element has an equal probability to reside at any position inside an intron, regardless of the distance of the intron from the CDS start. Then, we ran 1,000 simulations in which we uniformly repositioned the functional elements within the introns. Compatible with our previous observations, in more than 95% of the cases the randomly positioned miRNAs and snoRNAs resided in their actual hosting intron, or in an intron closer to the CDS start. In contrast, in less than 1% of the cases a TFBS resided in its actual hosting intron or in an intron closer to the CDS start. We conclude that the tendency of TFBS-bearing introns to reside in introns that are close to the CDS start is not a simple outcome of the fact that 5′-most introns are longer. Rather, this is likely to be a result of the fact that many of the TFBS affect transcription initiation and are located in the vicinity of the transcription start site.

**Figure 2:**
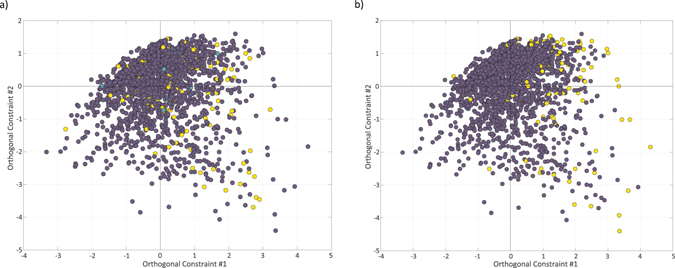
Fisher discriminant analysis for small RNA-bearing unique patterns in Dataset 1. **(a)** A scatter plot of all unique patterns, yellow: miRNA/snoRNA-bearing patterns, turquoise: miRNA/snoRNA-mixed patterns, and blue: miRNA/snoRNA-lacking patterns. The x-axis is the Fisher discriminant vector, the y-axis is used for visualization only, and represents the first principal component orthogonal to the Fisher discriminant vector. **(b)** Recoloring of the same scatter according to TFBS harboring state. Yellow: TFBS-bearing patterns, turquoise: TFBS -mixed patterns, and blue: TFBS-lacking patterns.

In order to further demonstrate that functional introns share common characteristics, regardless of their specific function, we performed Fisher discriminant analysis to separate miRNA/snoRNA-bearing patterns from miRNA/snoRAN-lacking ones (Fig. 2a). On this plot, introns that harbor small RNAs tend to have positive values of the Fisher discriminant vector. When coloring TFBS-bearing patterns, it is clearly evident that they too are characterized by high value of the Fisher discriminant vector (Fig. 2b, Supplementary Figure S1), suggesting that indeed functional introns have unique characteristics that are independent of the specific function.

### A semi-supervised probabilistic model allows the prediction of functional introns

Fisher discriminant analysis shows that functional introns tend to have high values of the Fisher discriminant vector, but that they are mixed with numerous non-functional introns (Fig. 2). This may be expected due to the inherent uncertainty in labeling introns as non-functional. When an intron is labeled functional, we are relatively confident that it is correctly labeled. However, when it is labeled non-functional, it may simply be the case that it is functional, but that the function is still unobserved, or simply of a type outside of the scope of the current study. We have therefore built a probabilistic model that takes this into account.

Let a unique pattern *i* be represented by the 13-dimensional feature vector *x*_*i*_, and let its functionality be labeled by ${y}_{i}\in \{1,0\}$, where 1 denotes a functional pattern and 0 denotes a non-functional one. We assume that the probability of a unique pattern *i* to represent a functional intron is given by the logistic function

1 $$p({y}_{i}=1|{x}_{i};\omega )=\,\frac{1}{1+{e}^{-{\omega }^{T}{x}_{i}+{\omega }_{0}}},$$

where ω_0_ and *ω* are parameters of the model (which we hereinafter group together as *ω*).

**Figure 3:**
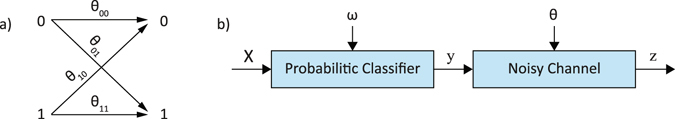
A schemes of the probabilistic model. **(a)** A diagram of the connections between the true label (left) and the observed label (right). **(b)** A diagram of logistic regression with noisy labels.

To incorporate the uncertainty in the labeling of unique patterns, we make a distinction between the actual label *z*_*i*_ and the true label *y*_*i*_, and treat *z*_*i*_ as a noisy version of *y*_*i*_. The relationship between the true and the noisy labels is described by ${\theta }_{ij}=p(z=j\,|y=i)$. The combined model is (Fig. 3):

2 $$p(z=j|x;\omega ,\theta )=\,\sum _{i}p(z=j|y=i\,;\theta )p(y=i|x;\omega )=\sum _{i}{\theta }_{ij}p(y=i|x;\omega )$$

Some of the *θ*_*ij*_’s may be determined from the particular nature of our problem. If a unique pattern is labeled as functional, we assume that it is indeed functional, which is formally expressed as ${\theta }_{01}=p(z=1|y=0)=0$. Therefore, ${\theta }_{00}=1-{\theta }_{01}=1$, and the sole undetermined *θ* value is *θ*_11_ (as *θ*_10_ = 1−*θ*_11_).

We used the Expectation-Maximization (EM) algorithm to learn the parameters of the model, $\omega$ and *θ*_11_, and then used (2) to compute the probability of each unique pattern to be functional. To determine which unique pattern corresponds to a functional intron we used a threshold *T* = 0.5, such that if $p({z}_{i}=j|{x}_{i};\omega ,\theta ) > T$ the unique pattern was classified as functional.

We used three different strategies to validate our model. First, we trained the model on Dataset 1, treating only the 134 small RNA-bearing unique patterns as functional, and tested the predictions of the model on the 88 TFBS-bearing unique patterns. We found that 82% of the TFBS-bearing patterns (72 out of 88) were correctly predicted as functional. Under our unique setup that the labeling of non-functional introns is uncertain, we cannot directly estimate the rate of false positives. To show that our success rate is not a result of a general tendency of our classifier to inflate the number of predicted functional patterns, we showed that the fraction of unique patterns predicted to be functional is higher among unique patterns labeled as TFBS-bearing than among those that are labeled as non-functional (P = 2.7·10^−11^, hypergeometric-test).

We wanted to understand what is special about the 16 TFBS-bearing patterns that we failed to detect. We found that all these patterns represent introns that are absent from plants, protists and fungi. Moreover, their LCA age is much younger than the average age of TFBS-bearing patterns and TFBS-lacking patterns too, for that matter (mean = 583 MYA). We have already discussed the fact that our approach is unable to detect recent acquisition of function, as the intron do not show unique evolutionary patterns. This is further reflected by the fact that the LOGLIKE values of all these patterns is significantly higher than the average value for TFBS-bearing patterns, and similarly their median distance from the CDS start is significantly greater than the value for TFBS-bearing patterns.

As a second validation, we have trained the model over Dataset 1 and tested it over Dataset 2. We correctly labeled 77% of the TFBS-bearing introns (581 out of 754). Again, to verify that our model does not inflate the number of predicted functional introns we performed a hypergeometric test (P = 3.1·10^−104^).

**Figure 4:**
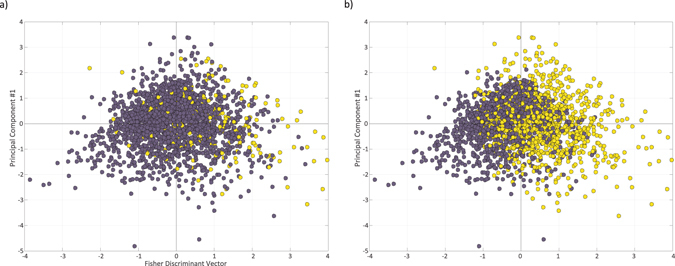
Fisher discriminant analysis over Dataset 1, treating all miRNA-, snoRNA- and TFBS-bearing patterns as functional, and the rest as non-functional. **(a)** A scatter plot of all unique patterns, yellow: function-bearing patterns, blue: function-lacking patterns. The x-axis is the Fisher discriminant vector, the y-axis is used for visualization only, and represents the first principal component orthogonal to the Fisher discriminant vector. **(b)** Recoloring of the same scatter according to model’s predictions. Yellow: predicted to be functional, blue: predicted to be non-functional.

As a third validation, we projected the predictions of the model onto the Fisher scatter plot. Figure 4 depicts the scatter plot when applying the Fisher discriminant analysis to Dataset 1, treating all miRNA-, snoRNA- and TFBS-bearing patterns as functional, and the rest as non-functional. In Fig. 4a, yellow dots mark those three groups of functional introns, showing that they tend to have high values of the Fisher discriminant vector. In Fig. 4b, yellow dots mark those patterns predicted as functional by our model. As expected, most of the patterns predicted as functional obtain high values of the Fisher discriminant vector. Interestingly, many patterns are still predicted as functional despite of having low values of the Fisher discriminant vector, demonstrating the limitations of the linear Fisher discriminant analysis.

Due to the sensitivity of the EM algorithm to the initial value of the estimated parameters, and due to the fact that it is not guaranteed to converge to the global maximum, we repeated the model training 100 times over each data set using random parameter initialization, and computed for each estimated parameter its mean, standard deviation (STD), and coefficient of variation (mean/STD). The results show the robustness of the training procedure, with the values of the estimated parameters insensitive to the initialization of the algorithm (Supplementary Table S2). Further, the estimated parameters were remarkably close even when the model was trained on different data sets (Supplementary Table S1, Supplementary Figure S2).

### The majority of functional introns are still unknown to be functional

From the estimated model parameters we wished to compute two values that are of great importance to our biological understanding of functional introns. The first is ${\theta }_{11}=\Pr ({z}_{i}=1|{y}_{i}=1)$, which is the probability of an intron to be observed as functional given that it is indeed functional. This is simply the fraction of functional introns that are known to be functional. The second is ${P}_{10}={\rm{\Pr }}({y}_{i}=1|{z}_{i}=0)$, which is the probability of an intron to be functional given that it is not labeled as one. This is simply the fraction of functional introns from those labeled as non-functional.

After training the model over our two data sets, we obtained that *θ*_11_ is in the range 0.17–0.22, suggesting that roughly 80% of the functional introns are yet to be found (Supplementary Figure S3a, Supplementary Table S3). *P*_10_ was estimated to be between 0.36–0.4, meaning that about 38% of the introns are potentially functional (Supplementary Figure S3b, Supplementary Table S3). This number is likely an overestimate, as the training is done over a set of highly conserved genes for which orthologs can be identified across the eukaryotic domain.

### Feature contribution to the prediction of functional introns

We next wished to evaluate the respective contribution of each of our 13 features to the predictor, and to test whether it is possible to reduce the number of features without significantly reducing the performance. To tackle these questions, we have used a battery of techniques. First, we computed the mutual information between each feature and the predicted functional patterns, with the understanding that high mutual information suggests high dependence between the predicted labels and the feature. The binary features IN_PLANT and IN_PROTIST display the highest mutual information values, followed by LOGLIKE and LCA_AGE (Supplementary Tables S4 and S5).

Second, we have tested the performance of the model using only 12 features. This was repeated 13 times, each time leaving another feature out of the analysis. We defined performance as the sum of square distances between the model predictions (Equation 2) using the 12 features and using the full set of features. The higher this distance is, the worse the performance without the omitted feature, and the greater is the importance of this feature. The highest distance was obtained for median(POSITION), followed by LCA_AGE and IN_PLANT (Supplementary Tables S4 and S5).

A third technique, related to the second, was to repeat the analysis for each feature separately. We defined performance as the sum of square distances between the model predictions when using a single feature and using the full set of features. The lower this distance is, the better the performance with this feature alone.The feature that performed better than all others in this analysis was median(POSITION), followed by LCA_AGE and IN_PLANT (Supplementary Tables S4 and S5).

Finally, we implemented a sequential features selection scheme. In each iteration the pair of features that contributed least to the performance was removed, and then the single feature with the highest contribution to the performance from among those already removed, was put back in the set. Compatible with the previous techniques, the last feature to remain after 12 iterations was median(POSITION), preceded by LCA_AGE and SANKOFF_G1L3 (Supplementary Tables S6 and S7).

### Enrichment of active genomic segments within introns predicted to be functional

We trained our model on Dataset 1 and computed for each human intron in Dataset 2 its probability to be functional. We then grouped the introns into those that are known to be functional (FUNC), those that are predicted to be functional by our model (PRED-FUNC), and those that are predicted to be non-functional (PRED-NON-FUNC). We wanted to compare these groups in terms of the density of functional regulatory elements they harbor. To this end, we used chromatic states as inferred by the ENCODE project. These states were determined based on the combination of two techniques (ChromHMM and Segway) which identify functional genomic segments based on experimental data that marks open chromatin, certain histone modifications, and transcription factor binding. Their data are available for six human cell types (GM12878, K562, H1-hESC, HeLa-S3, HepG2, and HUVEC), and is used to predict seven states: promoter region including TSS; promoter flanking region; enhancer; weak enhancer or open chromatin cis regulatory element; CTCF-rich element; transcribed region; and repressed or low activity region^20–23^. All the first six states correspond to active chromatin.

We crossed these data with our human introns, using the first six chromatin activity states. If at least one active segment was fully contained within an intron, this intron was marked as ‘active’. If no active segment overlapped an intron, is was marked as ‘inactive’. As the density of active segments within an intron is likely to depend on its length and GC content, we divided our introns into sixteen (4 × 4) bins (Supplementary Table S8). Only four bins contained introns from all three groups, FUNC, PRED-FUNC and PRED-NON-FUNC. In each of these four bins, we used Benjamini-Hochberg corrected *χ* ^2^-test to check whether the proportion of ‘active’ introns is different across the three groups of introns (Table 4). Whereas in most bins no significant difference between the intronic groups was found, the shortest and most GC-poor introns (length 0 to 11,000 bp, GC content between 0.25 to 0.5) showed higher proportion of active genomic segments in FUNC, followed by PRED-FUNC, and only then PRED-NON-FUNC (Table 4, Supplementary Table S9). The higher proportion of active segments in FUNC comes with no surprise, as we filtered the TFBS we used in compiling our datasets by being in active segments. However, the significantly higher proportion of PRED-FUNC compared to PRED-NON-FUNC shows that indeed introns predicted to be functional by our model have higher proportion of active genomic segments in comparison to introns that are not predicted to be functional by our model.

**Table 4:** Proportions of introns containing active genomic segments. Introns are separated to bins by their length and GC content.

	Bin 1	Bin 2	Bin 3	Bin 4
Intron Length (bp)	0–110000	0–110000	0–110000	110000–220000
GC content	0.25–0.5	0.5–0.75	0.75–1.0	0.25–0.5
**Proportions (percentage in parentheses)**				
Functional	399/424 (94.10)	265/303 (87.46)	27/29 (93.10)	3/3 (100.00)
Predicted to be functional	2249/2531 (88.86)	906/1082 (83.73)	10/15 (66.67)	4/7 (57.14)
Not predicted to be functional	6350/7383 (86.01)	2060/2457 (83.84)	20/27 (74.07)	4/9 (44.44)
Χ^2^ p-value	0*	0.2483	0.0664	0.2403
Significant pairs by Tukey’s test	FUNC vs. PRED-NON-FUNC, PRED-FUNC vs. PRED-NON-FUNC, FUNC vs. PRED-FUNC	—	—	—

Only bins with representatives of all three intronic groups (FUNC, PRED-FUNC, PRED-NON-FUNC) are shown. *χ* ^2^ p-values are before correction. Values marked with * are significant after correction for multiple hypotheses.

### Genes containing functional introns are linked to splicing and to mRNA processing

We next wanted to test whether genes hosting functional introns (known or predicted) are enriched with certain biological functions. To this end, we generated three list of genes. The first list contains all genes hosting at least one known functional intron. The second list contains all genes that are not present in the first list and that host at least one intron predicted to be functional by our model. The third list contains all other genes.

**Figure 5:**
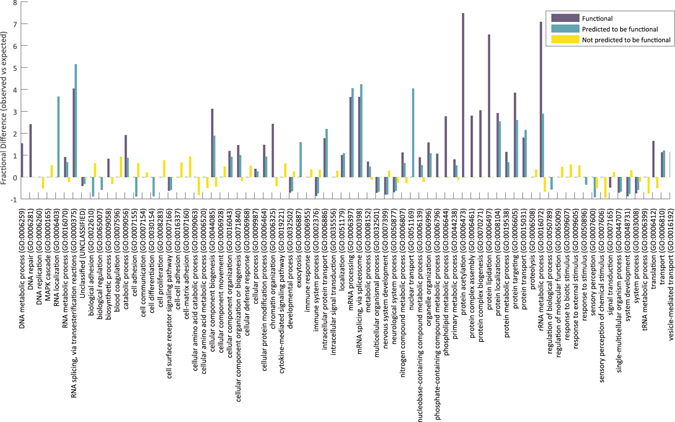
Comparison of fractional differences over GO terms of biological processes. In blue: hosting genes of functional introns, turquoise: hosting genes of introns predicted to be functional, yellow: hosting genes of introns not predicted to be functional.

Using the PANTHER gene ontology tool^27^, we tested each list for enrichment in biological processes against a background of all human genes (Supplementary Tables S10–S12). The fractional difference of each list over the unified list of 76 GO terms (Fig. 5) shows that genes that host functional introns, whether known or predicted, tend to be enriched/depleted with the same terms, in contrast to genes that harbor non-functional introns. This is also evident from the distance between the vectors of fractional differences between the three groups of genes (Supplementary Table S13). The most enriched terms in the list of genes that harbor introns predicted as functional are splicing and mRNA processing. These terms are also highly enriched in the list of genes that harbor introns that are known to be functional, but are entirely absent from the other genes.

The enrichment for splicing and mRNA processing may be attributed in part to the fact that we have focused on introns with embedded functional DNA elements whose process may be related to splicing and other pre-mRNA processes. However, this should affect much less genes with predicted functional introns, for which we see the same enrichment in biological processes.

### Functional introns tend to reside closer to the CDS start

**Figure 6:**
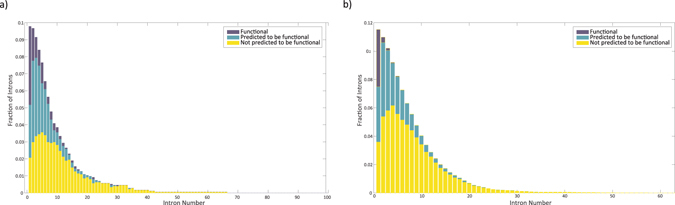
Distribution of functional introns (known or predicted) by intron number on **(a)** Dataset 1 and **(b)** Dataset 2. Blue: known functional introns, turquoise: introns predicted to be functional, yellow: introns predicted to be non-functional.

We have seen previously that functional introns tend to reside in the beginning of the coding sequence, especially if they harbor TFBS. We therefore tested whether introns that are predicted to be functional show the same tendency. As expected, these introns indeed tend to reside almost exclusively in the beginning of the CDS (Fig. 6).

### Partially-functional unique patterns show similar characteristics to non-functional unique patterns

Until now, unique patterns that were associated with multiple introns that some are known to be functional and some are not, were excluded from the analysis. We now wished to test whether the model tends to associate them with function or not. Surprisingly, only about 22% of these patterns were predicted to be functional, less than the overall average fraction of functional patterns predicted by the model. To further explore this, we computed for each partially-functional pattern the fraction of times in which it is associated with a functional intron. This fraction ranges from 0.0345 to 0.5, with an average of 0.348. We expect that partially-functional patterns predicted to be functional will display a higher fraction. Indeed, in partially-functional patterns that are predicted to be functional the mean fraction (0.422) is significantly above the average (P = 0.003).

## Discussion

In this work we developed a model that computes the probability of a human intron to be functional. Based on a comparative study of gene intron-exon architecture, we characterized introns by 13 features that mostly describe their evolutionary histories. Importantly, we do not look at intronic properties that are considered more evolutionary volatile, such as their sequence or length. Nor do we examine properties that characterize genes (like expression level), as the same pattern may be found in multiple genes. We showed that functional introns are characterized by a similar set of features, confirming the hypothesis that functional introns display lower loss rates and higher positional conservation, reflected in lower log-likelihood of the pattern, and in dense presence within certain clades (Table 3). An ancient origin of the function is in fact a requirement for our classifier to work, as it relies on evolutionary signatures that need time to be built. Indeed, we described a few examples of introns that acquired function only recently, and could not be detected by our algorithm.

Both small RNA-bearing introns and TFBS-bearing introns tend to reside closer to the 5′-end of the CDS (Table 3). We showed that while for small RNA-bearing introns this could be explained by the longer first introns, TFBS-bearing introns tend to reside near the CDS start regardless of the intron length. This is compatible with the possible role of intronic TFBS in regulating transcription initiation and in triggering alternative transcription start sites^2^.

A limitation of this study is that all the analyzed functional introns harbor a functional element in their sequence. We would have liked to include functional introns whose function stems from other features of the intron, like its location along the transcript or the mere fact that it had gone through splicing. However, there are no large-scale databases from which such information can be extracted, hindering our ability to carry out large-scale analyses. Nevertheless, the introns in this work harbored different kinds of functional elements, and still our classifier performed well on all these kinds regardless of what kinds it was trained upon. For example, when trained only on the small RNA-bearing introns of Dataset 1, the classifier successfully labeled 82% of the TFBS-bearing introns as functional. Similarly, when trained over Dataset 1, the classifier correctly labeled as functional 77% of the TFBS-bearing patterns in Dataset 2.

We examined the contribution of each feature to the performance of our classifier. It is difficult to assess each feature separately because of the features are inter-correlated and capture overlapping information. For this reason, we applied a battery of four different techniques that looked at this question from different angels. A few features consistently stood out as having the greatest contribution in all analyses, including features that measure the distance from the CDS start (median(POSTION)), the level of positional conservation (LOGLIKE, IN_PLANT) and the antiquity of the intron (LCA_AGE).

We build a statistical model that accounts for the asymmetrical state of knowledge regarding intron function. While an intron that harbors a functional element is highly likely to be functional, an intron that lacks such an element is not necessarily non-functional. It may well be functional by harboring a yet undetected functional element, by harboring a functional element not looked for in the current study, or by having a function that does not depend on an embedded functional element. Therefore, there is high certainty in labeling an intron as functional, but no certainty when labeling an intron as non-functional. We estimated the parameters of the model, and estimated that roughly 20% of the functional introns are labeled as such, and roughly 38% of the introns are functional. This number is expected to be an overestimation, because we had only taken highly conserved transcripts into the analysis. We expect that many introns within less conserved transcripts would lack function, and will thus reduce the above estimate.

We provide further validation of our predictions by showing that functional introns, as well as introns predicted to be functional, host more active genomic segments. In addition, genes that harbor functional introns, whether known to be functional or predicted to be one, tend to be enriched in GO terms of the same biological processes.

The model was trained on introns that are functional in humans, and therefore we expect our predictions to be most accurate for mammals. The ability to predict functional introns has obvious implications in fields such as studying the effect of mutations on human diseases and designing DNA constructs.

## Methods

### Gene architecture data

Gene exon-intron architecture data was downloaded from the JuncDB database^28^, that was updated to include new annotations from the Ensembl Genomes release 21^29^.

### Phylogenetic tree

Sets of orthologous genes were defined over 28 eukaryotic species. In addition to human, the sets include five mammals (*Pan troglodytes*, *Mus musculus*, *Pongo abelii*, *Ornithorhynchus anatinus*, *Monodelphis domestica*), a bird (*Gallus gallus*), two fish (*Danio rerio*, *Gasterosteus aculeatus*), two amphibians (*Anolis carolinensis*, *Xenopus tropicalis*), an insect (*Drosophila melanogaster*), three other invertebrates (*Caenorhabditis elegans*, *Schistosoma mansoni*, *Ciona savignyi*), four fungi (*Ustilago maydis*, *Saccharomyces cerevisiae*, *Neurospora crassa*, *Schizosaccharomyces pombe*), four plants (*Vitis vinifera*, *Arabidopsis thaliana*, *Physcomitrella patens*, *Oryza sativa*), and five protists (*Plasmodium falciparum*, *Dictyostelium discoideum*, *Leishmania major*, *Phaeodactylum tricornutum*, *Phytophthora infestans*). The phylogenetic tree that spans these 28 species was taken from our previous work^16^, with the exception that it has been modified to reflect the Ecdysozoa topology^30, 31^, which is now the commonly accepted alternative to the Coelomata topology^32, 33^ (Supplementary Figure S4, generated using iTOL^34, 35^). The modified topology was taken from The Tree of Life Web Project (http://tolweb.org/) and from NCBI^36^, and the updated divergence times were estimated using Time Tree^37^.

### Data sets of functional introns

The starting point of our previous work was a list of 450 human transcripts that host functional introns, where the intron function comes about from embedded miRNA or snoRNA elements^16^. Limiting the analysis to transcripts in which the functional introns are within the coding sequence (CDS), and crossing the data with the JuncDB database, we obtained 391 sets of orthologous transcripts over 28 species, in which the human transcript harbors at least one functional intron. In order to identify additional human functional introns, where the function stems from factors other than embedded small RNAs, we have extended our data set in two ways.

#### Dataset 1

First, we looked at all the introns in the above 450 human transcripts, and queried for the presence of intronic transcription factor binding sites (TFBS), using TFBS positions from the MotEvo database^38^. Out of the 450 transcripts, 306 were found to harbor TFBS-bearing introns within their CDS. Combining this with our previous data set of small RNA-bearing introns, we ended up with a set of 404 orthologous groups whose human transcript harbors at least one functional intron. To avoid a bias towards functional introns, we screened the Ensembl Genomes database (release 21) for transcripts whose introns are not known to contain small RNAs or TFBS, or to be otherwise functional. This added 56,957 human transcripts. Ensembl Compara API^39^ was used to search for orthologs of these transcripts in the other 27 species, resulting in 2,017 sets of orthologs. Obviously, not all 27 species have representatives in all the sets, and to avoid excessive amounts of missing data we removed from the data sets of orthologs with representatives from less than 13 species. This left 367 sets of orthologs. Altogether, Dataset 1 consist of 771 sets of orthologs: in 404 of the sets the human transcript is known to harbor a functional intron, and in 367 sets the human transcript lacks a known functional intron.

#### Dataset 2

In order to build a larger data set, we queried the MotEvo database again, this time against the complete Ensembl human transcripts data set, omitting only the transcripts who partake in Dataset 1. This resulted in 65,064 human transcripts harboring TFBS-bearing introns. Using Compara API, we have searched orthologs for these transcripts in the remaining 27 species, and formed a set of 8,661 orthologous groups in which the human transcript harbors a TFBS-bearing intron within the CDS, and in which there are at least five orthologous transcripts in other species. Among transcript variants that share introns and are members in different set of orthologs, we left in the analysis only the set with the highest number of representatives from other species.

#### Intron presence-absence patterns

For both data sets, we generated the multiple sequence alignments of the orthologous groups at the mRNA level. First, we used MUSCLE^40^ to align the protein products of the transcripts in each group, and then used Matlab’s *seqinsertgaps* function to properly align the mRNA. The aligned mRNA was then translated to a ternary representation; positions just before exon-exon junctions were denoted by 1, gaps and missing data were denoted by 2, and all other positions were denoted by 0. Each position across the alignment is an absence-presence pattern of introns. Patterns consisting of 45% or more missing values were filtered out, as well as patterns lacking 1’s. In addition, as the analysis is centered on humans, we filtered out patterns with 0’s or 2’s in human. TFBS that were not located in active ENCODE segments^20–23^ were filtered out as well.

### Statistical tests

Mann–Whitney U-test, and t-test with unknown and equal variance were used to test whether a distribution of a feature is different in function-bearing patterns. Tukey’s multiple comparison test for proportions was used to test whether there were significant dissimilarities between the proportions of introns bearing ENCODE active states across the three groups: functional introns, introns predicted to be functional, and introns predicted to be non-functional^41^.

### Fisher discriminant analysis

In order to avoid the use of excessively dependent features, we first applied principal component analysis to the data, and then used Fisher discriminant analysis on the data as represented by the first seven principal components.

### EM algorithm

Given *n* unique patterns *x*_1_,*x*_2_,…,*x*_*n*_ and their corresponding binary labels *z*_1_,*z*_2_,…*z*_*n*_, which are a noisy version of the hidden true labels *y*_1_,*y*_2_,…*y*_*n*_, the log likelihood of the model is

3 $$l(\omega ,\theta )=\sum _{t}\,\mathrm{log}\,p({z}_{t}|{x}_{t};\omega ,\theta )=\sum _{t}\,\mathrm{log}\,\sum _{i}p({z}_{t}|{y}_{t}=i;\theta )\,p({y}_{t}=i|{x}_{t};\omega ).$$

We define *c*_*ti*_ to be an estimation of the hidden label *y*_*t*_ given the features vector *x*_*t*_ and the noisy label z_t_, based on the current values of the parameters, *ω*_0_ and *θ*_0_,

4 $${c}_{ti}=p({y}_{t}=i|{z}_{t},{x}_{t};{\omega }_{0},{\theta }_{0}).$$

Then, the EM auxiliary function is:

5 $$\begin{array}{rcl}Q(\omega ,\theta ,{\omega }_{0},{\theta }_{0}) & = & \sum _{t}\,\mathrm{log}\,p({z}_{t},{y}_{t}|{x}_{t};\omega ,\theta )\\  & = & \sum _{t}\sum _{i=0,1}{c}_{ti}(\mathrm{log}\,p({z}_{t}|{y}_{t}=i;\theta )+\,\mathrm{log}\,p({y}_{t}=i|{x}_{t};\omega ))\\  & = & {L}_{1}(\theta )+{L}_{2}(\omega ).\end{array}$$

#### E-step

For *t* = 1,…,*n* and $i\in \{0,1\}$ we compute:

6 $${c}_{ti}=p({y}_{t}=i|{z}_{t},{x}_{t};{\omega }_{0},{\theta }_{0})=\,\frac{{\theta }_{0}(i,{z}_{t})p({y}_{t}=i|{x}_{t};{\omega }_{0})}{{\sum }_{k}{\theta }_{0}(k,{z}_{t})p({y}_{t}=k|{x}_{t};{\omega }_{0})}\,.$$

#### M-step

Based on the auxiliary function above, in order to find the updated value of $\theta$ it is sufficient to maximize

7 $${L}_{1}(\theta )=\sum _{t}\sum _{i,j}{1}_{\{{z}_{t}=1\}}{c}_{ti}\,\mathrm{log}\,{\theta }_{ij}.$$

Therefore, the updated value of $\theta \,\,$is:

$${\theta }_{ij}=\,\frac{{\sum }_{t}{c}_{ti}{1}_{\{{z}_{t}={\rm{j}}\}}}{{\sum }_{t}{c}_{ti}}\,i\in \{0,1\},\,\,j\in \{0,1\}.$$

By our assumptions regarding the nature of the labeling, *θ* can be reduced to *θ*_11_, therefore:

8 $$\theta ={\theta }_{11}=\frac{{\sum }_{t}{c}_{t1}{1}_{\{{z}_{t}=1\}}}{{\sum }_{t}{c}_{t1}}.$$

To find the updated value of *ω*, it is sufficient to maximize

9 $${L}_{2}(\omega )=\sum _{t}\sum _{i=0,1}{c}_{ti}\,\mathrm{log}\,p({y}_{t}=i|{x}_{t};\omega )=\sum _{t}{c}_{t0}\,\mathrm{log}\,p({y}_{t}=0|{x}_{t};\omega )+{c}_{t1}\,\mathrm{log}\,p({y}_{t}=1|{x}_{t};\omega ).\,$$

This is a weighted version of the cost function of a logistic regression. To find the maximum value, we can use gradient ascent, where the first derivative is

10 $$\frac{d{L}_{2}(\omega )}{d\omega }=\,\sum _{t}{x}_{t}({c}_{t1}-p({y}_{t}=1|x;\omega )),$$

and the second derivative is

11 $$\frac{{d}^{2}{L}_{2}(\omega )}{{d}^{2}\omega }=-\sum _{t}p({y}_{t}=1|x;\omega )\,p({y}_{t}=0|x;\omega ){x}_{t}{x}_{t}^{T}.$$

The second derivative is actually a Hessian matrix which is negative semi-definite for all *ω*, meaning this optimization problem is concave and therefore gradient ascent can be used in order to find the global maximum of *L*_2_(ω). Therefore, for iteration *n* and learning rate τ,

12 $${\omega }_{n+1}={\omega }_{n}+\tau \frac{d{L}_{2}({\omega }_{n})}{d\omega }.$$

### Mutual information

In order to evaluate the contribution of each feature to the prediction of functional introns, we computed for each feature the mutual information, defined as

13 $$I({X}_{i};Y)=\,\sum _{y}\sum _{{x}_{i}}P({x}_{i},y){\rm{l}}{\rm{o}}{\rm{g}}\,\frac{P({x}_{i},y)}{P({x}_{i})P(y)},$$

where *X*_*i*_ is the *i*’th feature, and *Y* is the predicted binary label.

### Sequential Features Selection

In order to find the smallest set of features needed for classification with minimal effect on performance, we have implemented a sequential ‘remove two add one’ features selection algorithm^42^. In each iteration, the pair of features that affects the performance the least is removed (added to the “rejected” set). Then, the rejected feature that contributes most to the performance is added back. For each set of features $F$, the performance is defined as

14 $${\rm{Performance}}(F)=\sum _{p}^{|patterns|}{(P{r}_{F}(p)-P{r}_{0}(p))}^{2},$$

where *Pr*_0_(*p*) denotes the probability of pattern *p* to be functional based on the full set of features, and *Pr*_F_(*p*) denotes the probability of pattern *p* to be functional based on the subset *F*.

## Electronic supplementary material

Supplementary Figures and Tables
