# Supplementary material for: Identification of introns harboring functional sequence elements through positional conservation
Source: Sci Rep. 2017 Jun 23;7:4201. doi: 10.1038/s41598-017-04476-0 (PMC5482813; doi:10.1038/s41598-017-04476-0)
Supplement: Supplementary file 1 — Supplementary Figures and Tables [file 41598_2017_4476_MOESM1_ESM.pdf]

Supplementary Files (Figures S1-S4, Tables S1-S13)

Identification of introns harboring functional sequence elements through positional conservation

Michal Chorev, Alan Joseph Bekker, Jacob Goldberger, Liran Carmel

Supplementary Files (Figures S1-S4, Tables S1-S13)

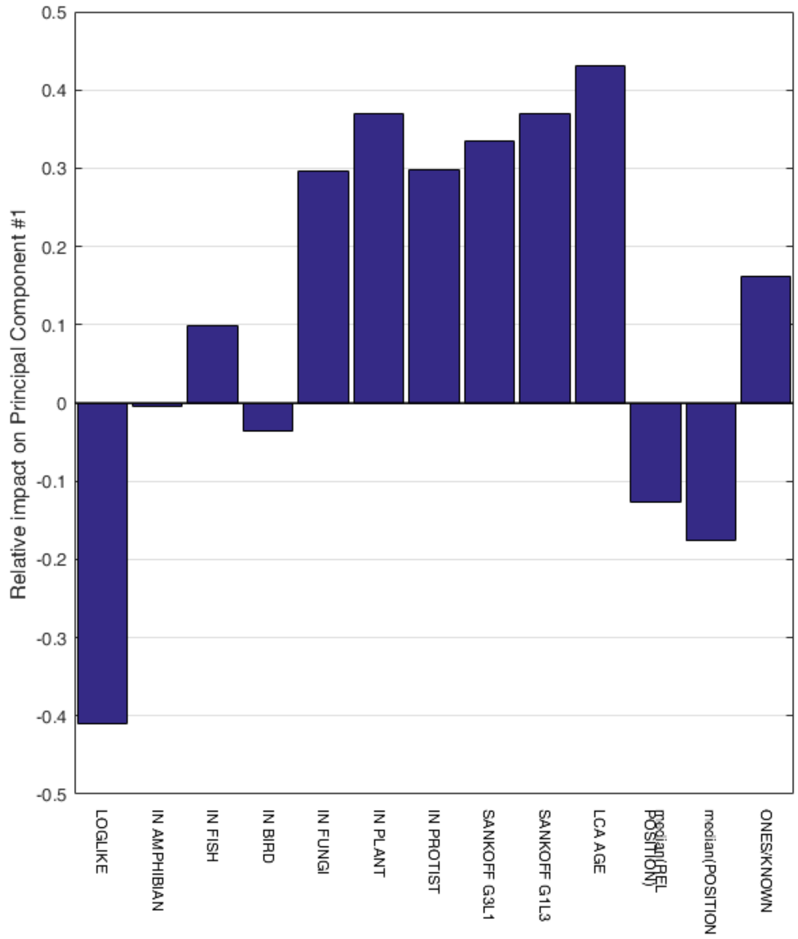

Figure S1. Loadings of the Fisher discriminant vector for small RNA-bearing unique patterns in Dataset 1

Supplementary Files (Figures S1-S4, Tables S1-S13)

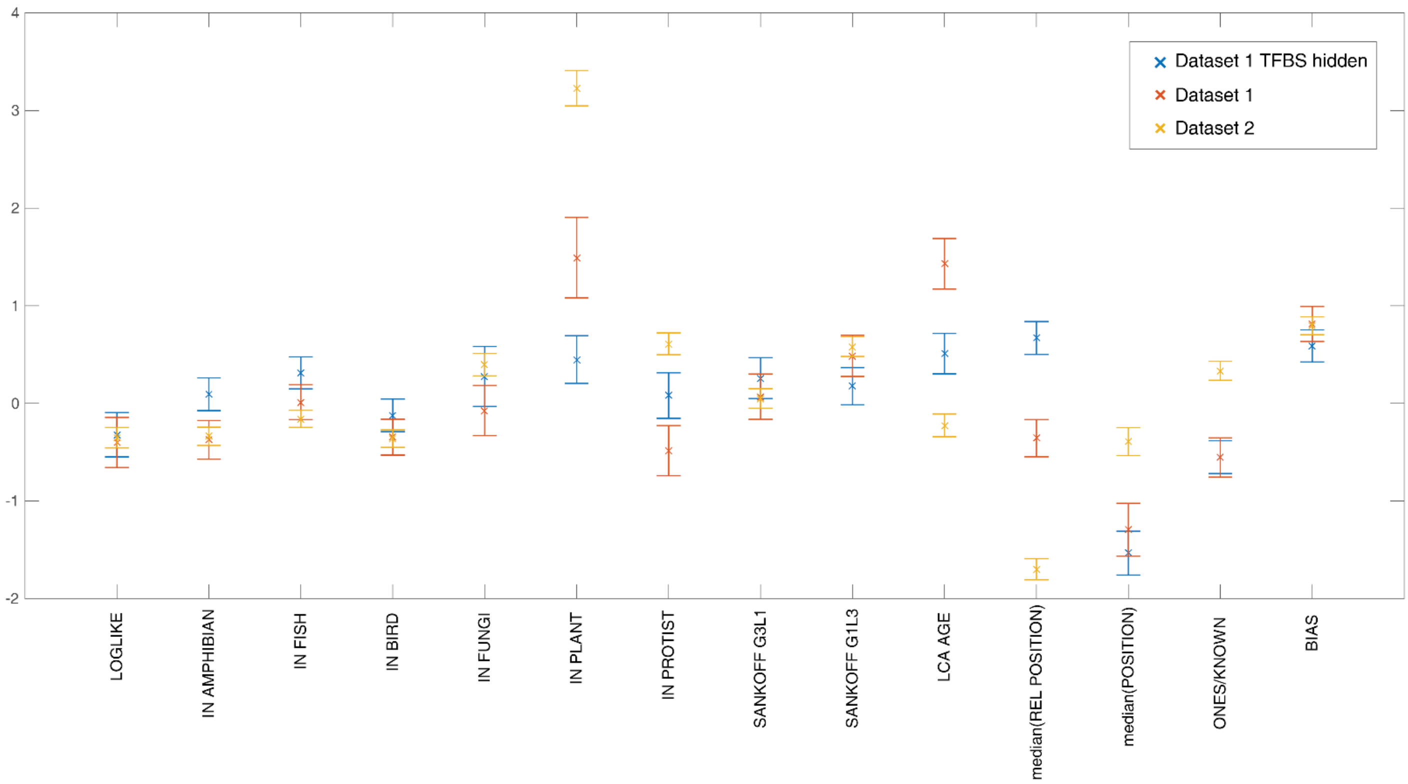

Figure S2. A plot of the weights estimations on all datasets

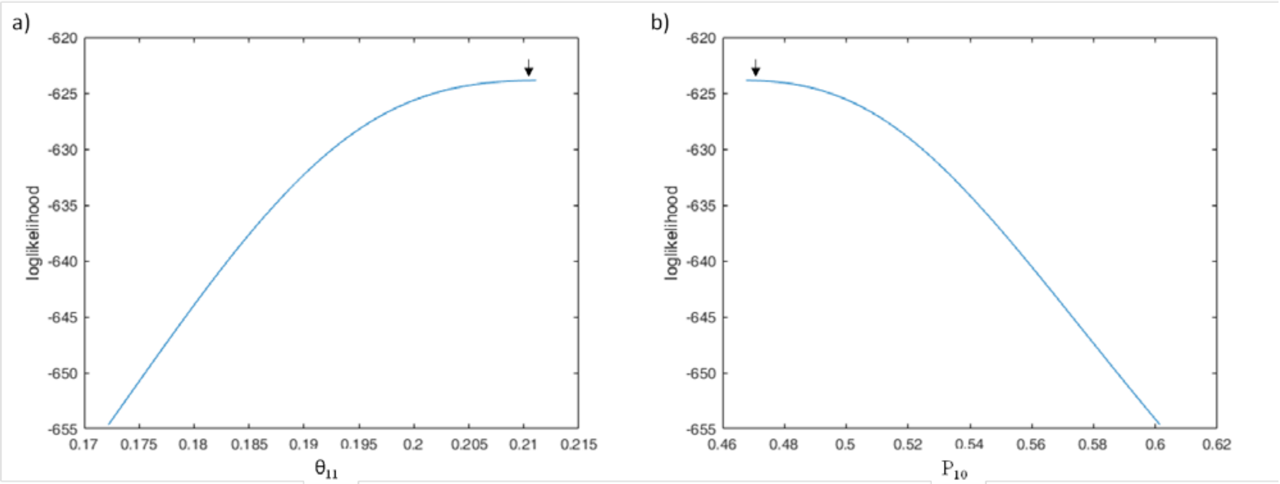

Figure S3. Log likelihood of the model as a function of parameter values, computed over Dataset 1. (a) Log likelihood as a function of θ_{11}. The maximum is achieved for $\theta_{11} = 0.21$. (b) Log likelihood as a function of P_{10}. The maximum is achieved for $P_{10} = 0.47$. The arrows mark the maximum log likelihood, after its decline the run was stopped.

Supplementary Files (Figures S1-S4, Tables S1-S13)

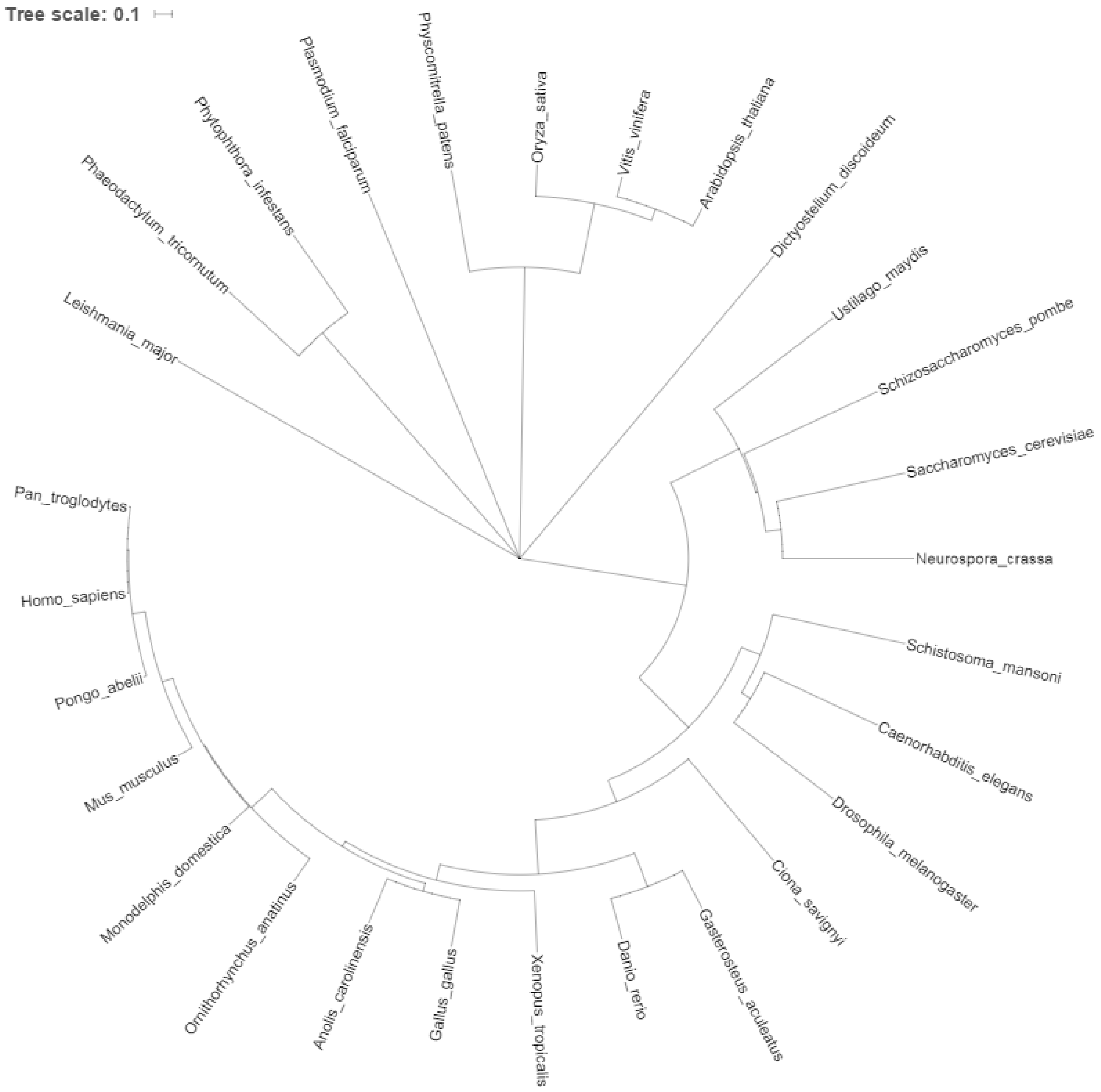

Figure S4. The phylogenetic tree of the 28 species.

Supplementary Files (Figures S1-S4, Tables S1-S13)

	miRNA and snoRNA			*TFBS in Dataset 1*			*TFBS in Dataset 2*		
	Bearing	Lacking	P-value (U-test)	Bearing	Lacking	P-value (U-test)	Bearing	Lacking	P-value (U-test)
LOGLIKE	-17.48	-12.83	**0.00027**	-23.12	-12.76	**$3.17x10^{-16}$**	-21.87	-15.65	**$3.36x10^{-72}$**
ONES_KNOWN	0.47	0.52	**0.00563**	0.49	0.5	1	0.563	0.56	1
SANKOFF_G3L1	4	4	**$7.96x10^{-5}$**	5	4	**$5.12x10^{-12}$**	4	4	**$8.20x10^{-43}$**
SANKOFF_G1L3	2	2	**0.00194**	4	2	**$7.91x10^{-18}$**	4	2	**$2.83x10^{-83}$**
IN_AMPHIBIAN	1	1	1	1	1	**$4.47x10^{-7}$**	1	1	**$4.21x10^{-25}$**
IN_FISH	1	1	1	1	1	1	1	1	1
IN_BIRD	1	1	1	1	1	**$9.41x10^{-7}$**	1	1	**$2.02x10^{-29}$**
IN_FUNGI	0	0	0.071	0	0	**$1.03x10^{-6}$**	0	0	**$5.6x10^{-41}$**
IN_PLANT	0	0	0.183	0	0	**$2.12x10^{-7}$**	0	0	**0.0015**
IN_PROTIST	0	0	1	0	0	**$3.2x10^{-16}$**	0	0	**$1.38x10^{-24}$**
LCA_AGE	910	774	**0.000802**	910	774	**$1.54x10^{-7}$**	910	774	**$2.87x10^{-24}$**
MED_REL_POSITION	0.501	0.519	1	0.067	0.536	**$4.5x10^{-44}$**	0.096	0.518	**0**
MED_POSITION	498	1066.5	**$3.63x10^{-14}$**	75.5	1073	**$1.35x10^{-51}$**	108	822	**0**

Table S1. *Median of the 13 features over TFBS-, miRNA- and snoRNA- bearing and lacking unique patterns.*

Supplementary Files (Figures S1-S4, Tables S1-S13)

	DATASET 1			DATASET 2		
	MEAN	STD	MEAN/STD	MEAN	STD	MEAN/STD
**WEIGHTS**						
LOGLIKE	-0.405	0.071	-5.709	-0.317	0.041	-7.777
IN AMPHIBIAN	-0.389	0.023	-17.158	-0.346	0.009	-39.861
IN FISH	0.030	0.015	1.908	-0.183	0.022	-8.453
IN BIRD	-0.390	0.012	-31.525	-0.360	0.0168	-21.379
IN FUNGI	0.0308	0.017	1.817	0.321	0.077	4.139
IN PLANT	0.944	0.224	4.213	1.189	0.645	1.843
IN PROTIST	-0.021	0.199	-0.105	0.0179	0.382	0.047
SANKOFF G3L1	0.251	0.097	2.595	0.007	0.120	0.057
SANKOFF G1L3	0.504	0.024	21.185	0.636	0.055	11.460
LCA AGE	0.752	0.275	2.740	0.495	0.546	0.906
median(REL POSITION)	-0.352	0.035	-10.023	-1.679	0.025	-67.883
median(POSITION)	-1.394	0.050	-27.753	-0.391	0.011	-36.760
ONES/KNOWN	-0.354	0.063	-5.612	0.207	0.103	2.010
BIAS	0.628	0.038	16.619	0.238	0.182	1.303
$\boldsymbol{\theta_{11}}$	0.206	0.002	86.122	0.219	0.005	40.270
$\boldsymbol{P_{10}}$	0.483	0.007	69.359	0.415	0.013	32.358

***Table S2**. Mean, standard deviation and mean/std values of all parameters estimated by the model for Datasets 1 and 2.*

	DATASET 1	DATASET 2
**WEIGHTS**		
LOGLIKE	-0.400756916	-0.351903739
IN AMPHIBIAN	-0.374109692	-0.337717863
IN FISH	0.012102558	-0.158850153
IN BIRD	-0.346564009	-0.360754574
IN FUNGI	-0.073572378	0.396546779
IN PLANT	1.493438906	3.228479546
IN PROTIST	-0.484704701	0.610245331
SANKOFF G3L1	0.068517194	0.050432796
SANKOFF G1L3	0.486468784	0.581545921
LCA AGE	1.429686099	-0.225297043
MEDIAN(REL POSITION)	-0.356722261	-1.698680391
MEDIAN(POSITION)	-1.29482842	-0.390761598
ONES/KNOWN	-0.554213072	0.334475875
BIAS	0.813653381	0.794926246
$\boldsymbol{\theta_{11}}$	0.210329	0.222235
$\boldsymbol{P_{10}}$	0.469793141	0.406600935

***Table S3**. Parameters estimation when training over Dataset 1 and Dataset 2.*

Supplementary Files (Figures S1-S4, Tables S1-S13)

	Mutual Information	Leave One Out	One Feature
**LOGLIKE**	0.087189715	51.20262	2039.091693
**IN AMPHIBIAN**	0.040487775	68.79483	2269.637191
**IN FISH**	0.002293931	20.14408	2273.421413
**IN BIRD**	0.044216985	44.76877	2281.394162
**IN FUNGI**	0.111915656	21.48884	2207.15707
**IN PLANT**	0.156382809	294.4145	2054.271517
**IN PROTIST**	0.092433974	45.81754	2191.223979
**SANKOFF G3L1**	0.06845695	22.61786	2130.818207
**SANKOFF G1L3**	0.069217943	73.91225	2018.520345
**LCA AGE**	0.063409095	203.5738	2024.646474
**median(REL POSITION)**	0.061844542	44.4248	2064.259079
**median(POSITION)**	0.054941604	2232.838	1804.032844
**ONES/KNOWN**	0.018905206	57.36982	2569.39967

Table S4. *Testing the features contribution to prediction over Dataset 1 using mutual information, leave-one-feature-out, and leave-a-single-feature.*

	Mutual Information	Leave One Out	One Feature
**LOGLIKE**	0.076244488	501.8354	2740.832132
**IN AMPHIBIAN**	0.008112945	542.8761	2909.279737
**IN FISH**	3.8673E-05	503.0123	2939.765521
**IN BIRD**	0.010372304	461.792	2893.998719
**IN FUNGI**	0.089718308	477.1537	2829.208685
**IN PLANT**	0.180288591	362.0706	2786.18203
**IN PROTIST**	0.095107859	519.2821	2831.614885
**SANKOFF G3L1**	0.036366966	524.1204	2827.491533
**SANKOFF G1L3**	0.050373327	549.15	2679.397671
**LCA AGE**	0.064663841	715.6832	2722.518394
**median(REL POSITION)**	0.05116373	95.80819	2577.255945
**median(POSITION)**	0.033762926	578.14	2559.482836
**ONES/KNOWN**	0.01281204	505.2004	3106.708854

Table S5. *Testing the features contribution to prediction over Dataset 2 using mutual information, leave-one-feature-out, and leave-a-single-feature.*

Number of features	Selected features in the analysis
**12**	median(POSITION), LCA AGE, SANKOFF G3L1, LOGLIKE, IN AMPHIBIAN, IN BIRD, IN FUNGI, IN PLANT, IN PROTIST, SANKOFF G1L3, median(REL POSITION), ONES/KNOWN
**11**	median(POSITION), LCA AGE, SANKOFF G3L1, LOGLIKE, IN AMPHIBIAN, IN BIRD, IN PLANT, IN PROTIST, SANKOFF G1L3, median(REL POSITION), ONES/KNOWN
**10**	median(POSITION), LCA AGE, SANKOFF G3L1, LOGLIKE, IN AMPHIBIAN, IN BIRD, IN PLANT, SANKOFF G1L3, median(REL POSITION), ONES/KNOWN
**9**	median(POSITION), LCA AGE, SANKOFF G3L1, LOGLIKE, IN AMPHIBIAN, IN BIRD, IN PLANT, SANKOFF G1L3, ONES/KNOWN, IN BIRD
**8**	median(POSITION), LCA AGE, SANKOFF G3L1, LOGLIKE, IN AMPHIBIAN, IN PLANT, IN BIRD, median(REL POSITION)
**7**	median(POSITION), LCA AGE, SANKOFF G3L1, LOGLIKE, IN AMPHIBIAN, IN BIRD, IN PLANT
**6**	median(POSITION), SANKOFF G3L1, LOGLIKE, IN BIRD, IN PLANT, SANKOFF G1L3
**5**	median(POSITION), SANKOFF G3L1, LOGLIKE, IN BIRD, IN PLANT
**4**	median(POSITION), LOGLIKE, IN PLANT, LCA AGE
**3**	median(POSITION), LCA AGE, SANKOFF G3L1
**2**	median(POSITION), LCA AGE
**1**	median(POSITION)

***Table S6**. The features contribution to the prediction over Dataset 1 using sequential feature selection.*

Number of features left	Selected features for maximum performance
**12**	LOGLIKE, IN AMPHIBIAN, IN FISH, IN BIRD, IN FUNGI, IN PLANT, SANKOFF G3L1, SANKOFF G1L3, LCA AGE, median(REL POSITION), median(POSITION), ONES/KNOWN
**11**	LOGLIKE, IN AMPHIBIAN, IN FISH, IN BIRD, IN FUNGI, IN PLANT, SANKOFF G3L1, SANKOFF G1L3, LCA AGE, median(REL POSITION), ONES/KNOWN
**10**	IN AMPHIBIAN, IN FISH, IN BIRD, IN FUNGI, IN PLANT, SANKOFF G3L1, SANKOFF G1L3, LCA AGE, median(REL POSITION), ONES/KNOWN
**9**	IN FISH, IN BIRD, IN FUNGI, IN PLANT, SANKOFF G3L1, SANKOFF G1L3, LCA AGE, median(REL POSITION), median(POSITION)
**8**	IN FISH, IN BIRD, IN FUNGI, IN PLANT, SANKOFF G3L1, SANKOFF G1L3, LCA AGE, median(REL POSITION)
**7**	IN BIRD, IN FUNGI, IN PLANT, SANKOFF G3L1, SANKOFF G1L3, LCA AGE, median(REL POSITION)
**6**	IN BIRD, IN FUNGI, IN PLANT, SANKOFF G1L3, median(REL POSITION), ONES/KNOWN
**5**	IN BIRD, IN FUNGI, IN PLANT, SANKOFF G1L3, median(REL POSITION)
**4**	IN FUNGI, IN PLANT, SANKOFF G1L3, median(REL POSITION)
**3**	IN PLANT, SANKOFF G1L3, median(REL POSITION)
**2**	SANKOFF G1L3, LCA AGE
**1**	median(POSITION)

Table S7. *Testing the features contribution to prediction over Dataset 2 using sequential feature selection.*

Length / GC content	0-0.25	0.25-0.5	0.5-0.75	0.75-1.0
**0-11000**	119 (0;37;82)	10246 (436;3351;6459)	3815 (305;1488;2022)	71 (29;18;24)
**0-11000**	1 (0;1;0)	19 (4;7;8)	0	0
**0-11000**	0	4 (2;1;1)	0	0
**0-11000**	0	4 (1;1;2)	0	0

Table S8. *Intron binning by length and GC content. In parenthesis is a breakdown of the total numbers to FUNC (known functional introns), PRED-FUNC (introns predicted to be functional) and NON-FUNC (for all other introns).*

	Pair	Diff	SE	q Statistic	Critical Value	Significant
**Bin 1**	NON-FUNC vs. PRED-FUNC	2.055	0.431	4.766	3.310	yes
	NON-FUNC vs. FUNC	5.453	1.002	5.443	3.310	yes
	PRED-FUNC vs. FUNC	3.398	1.031	3.296	3.310	no

Table S9. *Tukey's multiple comparisons of proportions test over the number of occurrences of introns bearing ENCODE states in three groups of introns: functional, predicted to be functional, and not predicted to be functional.*

Supplementary Files (Figures S1-S4, Tables S1-S13)

PANTHER GO-Slim Biological Process	Homo sapiens - REFLIST (20814)	Genes List (894)	Expected	Over/under	Fold Enrichment	P-value
peroxisomal transport (GO:0043574)	18	8	0.77	+	> 5	3.44E-04
gluconeogenesis (GO:0006094)	14	6	0.6	+	> 5	8.58E-03
regulation of carbohydrate metabolic process (GO:0006109)	21	7	0.9	+	> 5	9.55E-03
protein lipidation (GO:0006497)	31	9	1.33	+	> 5	2.37E-03
rRNA metabolic process (GO:0016072)	115	26	4.94	+	> 5	4.09E-09
protein targeting (GO:0006605)	112	24	4.81	+	4.99	6.92E-08
RNA catabolic process (GO:0006401)	56	12	2.41	+	4.99	1.82E-03
protein localization (GO:0008104)	116	24	4.98	+	4.82	1.37E-07
mRNA processing (GO:0006397)	274	56	11.77	+	4.76	9.14E-19
RNA splicing, via transesterification reactions (GO:0000375)	132	25	5.67	+	4.41	3.53E-07
cellular amino acid catabolic process (GO:0009063)	53	10	2.28	+	4.39	2.84E-02
RNA splicing (GO:0008380)	135	25	5.8	+	4.31	5.49E-07
mRNA splicing, via spliceosome (GO:0000398)	183	33	7.86	+	4.2	3.18E-09
regulation of translation (GO:0006417)	148	25	6.36	+	3.93	3.25E-06
glycogen metabolic process (GO:0005977)	87	14	3.74	+	3.75	7.83E-03
nuclear transport (GO:0051169)	85	13	3.65	+	3.56	2.42E-02
cellular component biogenesis (GO:0044085)	310	46	13.32	+	3.45	2.65E-10
catabolic process (GO:0009056)	407	54	17.48	+	3.09	2.10E-10
protein transport (GO:0015031)	1082	135	46.47	+	2.9	8.25E-26
mitosis (GO:0007067)	367	45	15.76	+	2.85	1.93E-07
DNA repair (GO:0006281)	172	21	7.39	+	2.84	6.25E-03
translation (GO:0006412)	435	52	18.68	+	2.78	2.25E-08
intracellular protein transport (GO:0006886)	1052	124	45.19	+	2.74	2.12E-21
phospholipid metabolic process (GO:0006644)	182	21	7.82	+	2.69	1.38E-02
vesicle-mediated transport (GO:0016192)	895	97	38.44	+	2.52	5.27E-14
organelle organization (GO:0006996)	571	59	24.53	+	2.41	2.92E-07
DNA metabolic process (GO:0006259)	379	39	16.28	+	2.4	2.06E-04
exocytosis (GO:0006887)	258	26	11.08	+	2.35	1.80E-02
cellular component	1316	118	56.52	+	2.09	1.46E-11

organization or biogenesis (GO:0071840)						
nitrogen compound metabolic process (GO:0006807)	1099	94	47.2	+	1.99	7.67E-08
cellular protein modification process (GO:0006464)	1317	112	56.57	+	1.98	1.80E-09
cell cycle (GO:0007049)	1107	92	47.55	+	1.93	4.86E-07
protein metabolic process (GO:0019538)	2692	223	115.63	+	1.93	5.41E-20
carbohydrate metabolic process (GO:0005975)	573	47	24.61	+	1.91	6.37E-03
cellular component organization (GO:0016043)	1206	97	51.8	+	1.87	8.71E-07
transport (GO:0006810)	2473	192	106.22	+	1.81	8.54E-14
localization (GO:0051179)	2607	201	111.98	+	1.8	2.78E-14
phosphate-containing compound metabolic process (GO:0006796)	910	70	39.09	+	1.79	6.69E-04
protein phosphorylation (GO:0006468)	603	46	25.9	+	1.78	3.99E-02
nucleobase-containing compound metabolic process (GO:0006139)	3467	241	148.91	+	1.62	1.51E-12
primary metabolic process (GO:0044238)	6825	472	293.15	+	1.61	1.38E-32
metabolic process (GO:0008152)	8247	551	354.22	+	1.56	6.76E-38
RNA metabolic process (GO:0016070)	2360	150	101.37	+	1.48	1.78E-04
cellular process (GO:0009987)	6708	361	288.12	+	1.25	4.02E-05
Unclassified (UNCLASSIFIED)	8629	201	370.63	-	0.54	0.00E+00
regulation of transcription from RNA polymerase II promoter (GO:0006357)	1319	26	56.65	-	0.46	5.34E-04
developmental process (GO:0032502)	2456	45	105.49	-	0.43	5.50E-10
neurological system process (GO:0050877)	1064	19	45.7	-	0.42	9.74E-04
single-multicellular organism process (GO:0044707)	1636	27	70.27	-	0.38	2.32E-07
multicellular organismal process (GO:0032501)	1640	27	70.44	-	0.38	2.07E-07
system process (GO:0003008)	1296	21	55.67	-	0.38	1.01E-05
immune system process (GO:0002376)	1391	22	59.75	-	0.37	1.77E-06
nervous system development (GO:0007399)	823	8	35.35	-	0.23	4.92E-06
system development (GO:0048731)	1271	10	54.59	-	< 0.2	1.06E-11
ectoderm development (GO:0007398)	663	5	28.48	-	< 0.2	1.30E-05
mesoderm development (GO:0007498)	671	5	28.82	-	< 0.2	9.64E-06
sensory perception (GO:0007600)	455	3	19.54	-	< 0.2	8.95E-04

cell adhesion (GO:0007155)	579	3	24.87	-	< 0.2	7.71E-06
biological adhesion (GO:0022610)	606	3	26.03	-	< 0.2	2.67E-06

***Table S10**. PANTHER GO-slim biological process analysis for genes hosting known functional introns.*

Supplementary Files (Figures S1-S4, Tables S1-S13)

PANTHER GO-Slim Biological Process	Homo sapiens - REFLIST (20814)	Genes List (588)	Expected	Over/under	Fold Enrichment	P-value
RNA splicing, via transesterification reactions (GO:0000375)	132	19	3.73	+	> 5	3.21E-06
nuclear transport (GO:0051169)	85	12	2.4	+	5	1.75E-03
RNA splicing (GO:0008380)	135	19	3.81	+	4.98	4.55E-06
rRNA metabolic process (GO:0016072)	115	14	3.25	+	4.31	1.66E-03
mRNA splicing, via spliceosome (GO:0000398)	183	19	5.17	+	3.68	4.26E-04
mRNA processing (GO:0006397)	274	28	7.74	+	3.62	2.33E-06
DNA repair (GO:0006281)	172	17	4.86	+	3.5	2.73E-03
translation (GO:0006412)	435	39	12.29	+	3.17	1.20E-07
cellular component biogenesis (GO:0044085)	310	26	8.76	+	2.97	3.13E-04
protein transport (GO:0015031)	1082	78	30.57	+	2.55	1.52E-11
intracellular protein transport (GO:0006886)	1052	75	29.72	+	2.52	8.35E-11
DNA metabolic process (GO:0006259)	379	27	10.71	+	2.52	3.67E-03
vesicle-mediated transport (GO:0016192)	895	59	25.28	+	2.33	6.03E-07
cellular component organization or biogenesis (GO:0071840)	1316	76	37.18	+	2.04	8.72E-07
cellular component organization (GO:0016043)	1206	65	34.07	+	1.91	1.46E-04
protein metabolic process (GO:0019538)	2692	143	76.05	+	1.88	1.15E-11
cellular protein modification process (GO:0006464)	1317	65	37.21	+	1.75	2.51E-03
transport (GO:0006810)	2473	118	69.86	+	1.69	2.11E-06
localization (GO:0051179)	2607	119	73.65	+	1.62	2.04E-05
primary metabolic process (GO:0044238)	6825	287	192.81	+	1.49	1.51E-13
metabolic process (GO:0008152)	8247	342	232.98	+	1.47	2.09E-17
Unclassified (UNCLASSIFIED)	8629	160	243.77	-	0.66	0.00E+00
developmental process (GO:0032502)	2456	40	69.38	-	0.58	8.67E-03
regulation of nucleobase-containing compound metabolic process (GO:0019219)	1700	24	48.03	-	0.5	1.25E-02
immune system process (GO:0002376)	1391	17	39.3	-	0.43	7.32E-03
mesoderm development (GO:0007498)	671	5	18.96	-	0.26	2.95E-02
nervous system development (GO:0007399)	823	6	23.25	-	0.26	3.94E-03
system development (GO:0048731)	1271	9	35.91	-	0.25	1.06E-05

ectoderm development (GO:0007398)	663	3	18.73	-	< 0.2	1.68E-03
cell adhesion (GO:0007155)	579	1	16.36	-	< 0.2	2.46E-04
biological adhesion (GO:0022610)	606	1	17.12	-	< 0.2	1.17E-04

Table S11. *PANTHER GO-slim biological process analysis for genes hosting introns predicted to be functional by our model.*

Supplementary Files (Figures S1-S4, Tables S1-S13)

PANTHER GO-Slim Biological Process	Homo sapiens - REFLIST (20814)	Genes List (6540)	Expected	Over/under	Fold Enrichment	P-value
blood coagulation (GO:0007596)	168	94	52.79	+	1.78	3.88E-05
B cell mediated immunity (GO:0019724)	141	77	44.3	+	1.74	1.09E-03
cellular defense response (GO:0006968)	231	123	72.58	+	1.69	8.13E-06
immune response (GO:0006955)	518	267	162.76	+	1.64	4.33E-12
heart development (GO:0007507)	181	92	56.87	+	1.62	2.29E-03
response to external stimulus (GO:0009605)	378	189	118.77	+	1.59	2.68E-07
biological adhesion (GO:0022610)	606	290	190.41	+	1.52	1.24E-09
cell adhesion (GO:0007155)	579	277	181.93	+	1.52	3.82E-09
lipid transport (GO:0006869)	322	153	101.18	+	1.51	1.75E-04
cell-cell adhesion (GO:0016337)	391	184	122.86	+	1.5	2.67E-05
immune system process (GO:0002376)	1391	637	437.07	+	1.46	1.65E-18
nervous system development (GO:0007399)	823	371	258.6	+	1.43	2.45E-09
cell-cell signaling (GO:0007267)	633	284	198.9	+	1.43	1.01E-06
cellular component movement (GO:0006928)	476	212	149.56	+	1.42	1.44E-04
system development (GO:0048731)	1271	559	399.36	+	1.4	7.65E-13
ectoderm development (GO:0007398)	663	285	208.32	+	1.37	3.78E-05
proteolysis (GO:0006508)	719	308	225.92	+	1.36	1.64E-05
mesoderm development (GO:0007498)	671	272	210.84	+	1.29	4.95E-03
regulation of molecular function (GO:0065009)	1096	444	344.38	+	1.29	1.48E-05
response to stimulus (GO:0050896)	2170	877	681.84	+	1.29	3.64E-12
cell death (GO:0008219)	562	227	176.59	+	1.29	2.76E-02
death (GO:0016265)	566	228	177.84	+	1.28	3.09E-02
regulation of catalytic activity (GO:0050790)	1073	430	337.15	+	1.28	7.32E-05
developmental process (GO:0032502)	2456	983	771.7	+	1.27	6.89E-13
cell communication (GO:0007154)	3006	1198	944.52	+	1.27	8.25E-16
localization (GO:0051179)	2607	939	819.15	+	1.15	1.34E-03
transport (GO:0006810)	2473	879	777.05	+	1.13	1.52E-02
cellular process (GO:0009987)	6708	2373	2107.73	+	1.13	4.45E-10
primary metabolic process (GO:0044238)	6825	2011	2144.49	-	0.94	4.75E-02
Unclassified (UNCLASSIFIED)	8629	2505	2711.33	-	0.92	0.00E+00
nucleobase-containing compound metabolic process (GO:0006139)	3467	951	1089.37	-	0.87	3.60E-04
biosynthetic process	764	180	240.06	-	0.75	4.96E-03

(GO:0009058)						
nitrogen compound metabolic process (GO:0006807)	1099	249	345.32	-	0.72	3.10E-06
carbohydrate metabolic process (GO:0005975)	573	124	180.04	-	0.69	1.05E-03
chromatin organization (GO:0006325)	250	49	78.55	-	0.62	4.80E-02
DNA metabolic process (GO:0006259)	379	71	119.09	-	0.6	2.42E-04
regulation of translation (GO:0006417)	148	24	46.5	-	0.52	4.52E-02
DNA repair (GO:0006281)	172	26	54.04	-	0.48	3.67E-03
translation (GO:0006412)	435	65	136.68	-	0.48	9.74E-10
cellular component biogenesis (GO:0044085)	310	42	97.41	-	0.43	3.54E-08
cellular amino acid metabolic process (GO:0006520)	264	33	82.95	-	0.4	6.79E-08
DNA replication (GO:0006260)	161	20	50.59	-	0.4	1.77E-04
rRNA metabolic process (GO:0016072)	115	14	36.13	-	0.39	5.10E-03
tRNA metabolic process (GO:0006399)	82	8	25.77	-	0.31	9.52E-03
protein complex assembly (GO:0006461)	107	8	33.62	-	0.24	2.76E-05
protein complex biogenesis (GO:0070271)	108	8	33.93	-	0.24	2.17E-05
cellular amino acid catabolic process (GO:0009063)	53	3	16.65	-	< 0.2	1.18E-02
sensory perception of chemical stimulus (GO:0007606)	133	3	41.79	-	< 0.2	1.83E-12

Table S12. PANTHER GO-slim biological process analysis for genes hosting introns predicted to be non-functional by our model.

	Functional	Predicted functional	Other
Functional	0	9.52	16.03
Predicted functional		0	12.74
Other			0

***Table S13**. Pairwise standardized Euclidean distance between the vectors of fractional differences of GO terms over genes containing functional introns, genes containing predicted functional introns, and all other genes.*
